# The Korean undiagnosed diseases program phase I: expansion of the nationwide network and the development of long-term infrastructure

**DOI:** 10.1186/s13023-022-02520-5

**Published:** 2022-10-08

**Authors:** Soo Yeon Kim, Seungbok Lee, Hyewon Woo, Jiyeon Han, Young Jun Ko, Youngkyu Shim, Soojin Park, Se Song Jang, Byung Chan Lim, Jung Min Ko, Ki Joong Kim, Anna Cho, Hunmin Kim, Hee Hwang, Ji Eun Choi, Man Jin Kim, Jangsup Moon, Moon-Woo Seong, Sung Sup Park, Sun Ah Choi, Ji Eun Lee, Young Se Kwon, Young Bae Sohn, Jon Soo Kim, Won Seop Kim, Yun Jeong Lee, Soonhak Kwon, Young Ok Kim, Hoon Kook, Yong Gon Cho, Chong Kun Cheon, Ki-Soo Kang, Mi-Ryoung Song, Young-Joon Kim, Hyuk-Jin Cha, Hee-Jung Choi, Yun Kee, Sung-Gyoo Park, Seung Tae Baek, Murim Choi, Dong-Sung Ryu, Jong-Hee Chae

**Affiliations:** 1 Department of Genomic Medicine, Seoul National University Hospital, Seoul, Republic of Korea; 2 Department of Pediatrics, Pediatric Clinical Neuroscience Center, Seoul National University Children’s Hospital, National University College of Medicine, 101 Daehakro Jongno-gu, Seoul, 110-744 Republic of Korea; 3 Department of Pediatrics, Seoul National University Bundang Hospital, Seongnam, Republic of Korea; 4 Department of Pediatrics, Korea University Ansan Hospital, Ansan, Republic of Korea; 5 Division of Clinical Genetics, Department of Pediatrics, Seoul National University College of Medicine, Seoul, Republic of Korea; 6 Department of Pediatrics, SMG-SNU Boramae Hospital, Seoul, Republic of Korea; 7 Department of Laboratory Medicine, Seoul National University Hospital, Seoul, Republic of Korea; 8 Department of Pediatrics, Ehwa Womans University Mokdong Hospital, Ehwa Womans University College of Medicine, Seoul, Republic of Korea; 9 Department of Pediatric, Inha University College of Medicine, Inha University Hospital, Incheon, Republic of Korea; 10 Department of Medical Genetics, Ajou University Hospital, Ajou University School of Medicine, Suwon, Republic of Korea; 11 Department of Pediatrics, Chungbuk National University Hospital, Cheongju, Republic of Korea; 12 Department of Pediatrics, College of Medicine, Chungbuk National University, Cheongju, Republic of Korea; 13 Department of Pediatrics, School of Medicine, Kyungpook National University, Kyungpook National University Hospital, Daegu, Republic of Korea; 14 Departmentof Pediatrics, Chonnam National University Medical School, Gwangju, Republic of Korea; 15 Department of Laboratory Medicine, Jeonbuk National University Medical School, Jeonju, Republic of Korea; 16 Department of Pediatrics, Pusan National University Yangsan Hospital, Yangsan, Republic of Korea; 17 Department of Pediatrics, Jeju National University Hospital, Jeju, Republic of Korea; 18 School of Life Sciences, Gwangju Institute of Science and Technology, Gwangju, Republic of Korea; 19 Collage of Pharmacy, Seoul National University, Seoul, Republic of Korea; 20 School of Biological Sciences, Seoul National University, Seoul, Republic of Korea; 21 Division of Biomedical Convergence, College of Biomedical Science, Kangwon National University, Chuncheon, Republic of Korea; 22 Department of Life Sciences, Pohang University of Science and Technology, Pohang, Republic of Korea; 23 Department of Biomedical Sciences, Seoul National University College of Medicine, Seoul, Republic of Korea; 24 Theragen Bio Co., Ltd., Seongnam, Republic of Korea

**Keywords:** Rare disease, Undiagnosed disease program, Translational research, Data sharing

## Abstract

**Background:**

Phase I of the Korean Undiagnosed Diseases Program (KUDP), performed for 3 years, has been completed. The Phase I program aimed to solve the problem of undiagnosed patients throughout the country and develop infrastructure, including a data management system and functional core laboratory, for long-term translational research. Herein, we share the clinical experiences of the Phase I program and introduce the activities of the functional core laboratory and data management system.

**Results:**

During the program (2018–2020), 458 patients were enrolled and classified into 3 groups according to the following criteria: (I) those with a specific clinical assessment which can be verified by direct testing (32 patients); (II) those with a disease group with genetic and phenotypic heterogeneity (353 patients); and (III) those with atypical presentations or diseases unknown to date (73 patients). All patients underwent individualized diagnostic processes based on the decision of an expert consortium. Confirmative diagnoses were obtained for 242 patients (52.8%). The diagnostic yield was different for each group: 81.3% for Group I, 53.3% for Group II, and 38.4% for Group III. Diagnoses were made by next-generation sequencing for 204 patients (84.3%) and other genetic testing for 35 patients (14.5%). Three patients (1.2%) were diagnosed with nongenetic disorders. The KUDP functional core laboratory, with a group of experts, organized a streamlined research pipeline covering various resources, including animal models, stem cells, structural modeling and metabolic and biochemical approaches. Regular data review was performed to screen for candidate genes among undiagnosed patients, and six different genes were identified for functional research. We also developed a web-based database system that supports clinical cohort management and provides a matchmaker exchange protocol based on a matchbox, likely to reinforce the nationwide clinical network and further international collaboration.

**Conclusions:**

The KUDP evaluated the unmet needs of undiagnosed patients and established infrastructure for a data-sharing system and future functional research. The advancement of the KUDP may lead to sustainable bench-to-bedside research in Korea and contribute to ongoing international collaboration.

**Supplementary Information:**

The online version contains supplementary material available at 10.1186/s13023-022-02520-5.

## Background

Approximately 7000 types of rare diseases (RDs) have been genetically identified to date, and it has become easy for people living with rare disease (PLWRD) to access genetic testing and obtain a diagnosis as standard care [1, 2]. Unlike prior undiagnosed disease programs that focused on the diagnosis itself, recent studies have emphasized determining disease mechanisms and searching for therapeutic targets using tools such as data sharing, model organisms and supporting experiments, as well as identifying ultrarare diseases [3–6]. However, the clinical unmet needs of PLWRD still exist. There are still many genetically unidentified diseases and many nongenetic RDs often hard to distinguish from genetic RDs in practice. PLWRD account for approximately 8–10% of the general population, and their prompt diagnosis remains a challenge in many countries despite recent cost reductions and the easy accessibility of next-generation sequencing (NGS) [2]. Many national projects for RDs have been organized to support clinical needs and establish a nationwide registry and infrastructure [7–10].

The Korean Undiagnosed Diseases Program (KUDP) was launched as a pilot project in 2017 to support undiagnosed patients and initiate RD research. We reported the results of a pilot project, which indicated a 38.9% diagnostic yield from clinical protocols suitable for the Korean insurance system [11]. The Phase I main project continued for 3 years (2018–2020), focusing on advancing the workflow and establishing a long-term research platform. We tried to induce some changes in the medical insurance system of South Korea and apply an additional diagnostic algorithm for the remaining undiagnosed patients from the KUDP pilot project. Herein, we summarized the 3-year KUDP Phase I results and introduced the future directions of Korean RD research for undiagnosed patients.

## Results

### Patient characteristics

**Fig. 1:**
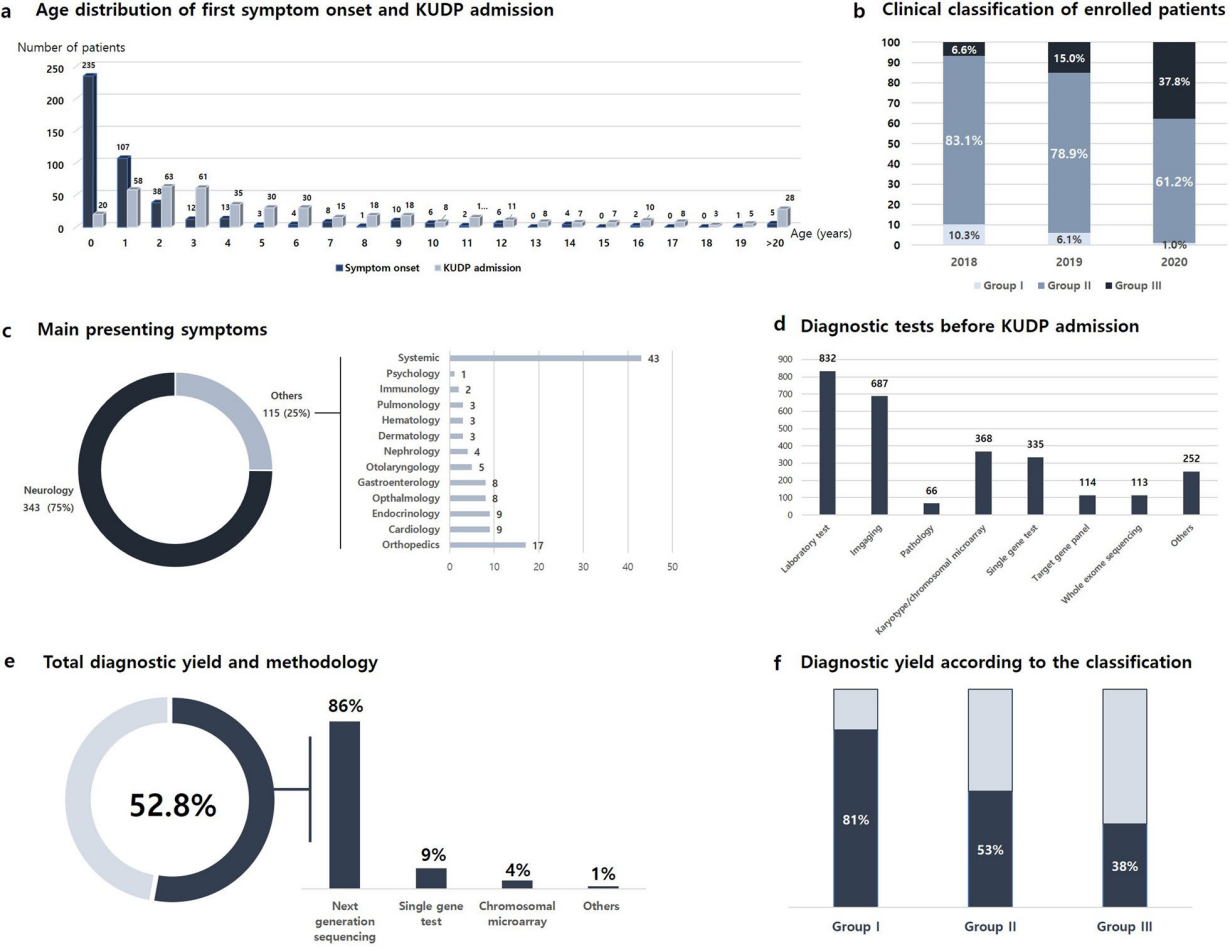
Summary of the clinical information and results. **a** The graph indicates the time gap between symptom onset and admission to the Korean Undiagnosed Diseases Program (KUDP). **b** The proportion of patients in Group III increased every year. **c** Neurological features were the most frequent presenting symptoms among the patients. **d** The enrolled patients underwent numerous diagnostic tests, including next-generation sequencing, before KUDP admission. **e** Final diagnoses were made for 52.8% of the patients by traditional tests and next-generation sequencing. **f** The diagnostic yield was dramatically different from group to group

Demographic and clinical information for the 458 enrolled patients is described in Table 1 and Fig. 1. Approximately one-third of the patients (157/458, 34.3%) presented their first symptoms before one month of age. Diagnostic evaluation by experts (at tertiary hospitals) was started within 6 months of symptom onset for 387 patients (84.5%). The median time between the first diagnostic evaluation and KUDP admission was 4.6 years (range, 0–37 years). Only 59 of 458 patients (12.9%) were enrolled in KUDP within 6 months of the first medical evaluation, and 131 patients (28.6%) had spent more than 5 years seeking a diagnosis before KUDP admission. The enrolled patients were classified into Group I (32 patients, 7.0%), Group II (353 patients 77.1%), or Group III (73 patients, 16.0%) based on the criteria described in the Methods section. The proportion of Group III patients increased each year, whereas that of Group I decreased. The main presentation was neurological symptoms in most patients (343/458, 74.9%) and simultaneous multisystemic features in 43 patients (9.4%). Multiple organ involvement was noted in 331 patients (72.3%). Patients visited an average of 2.1 tertiary hospitals (range 0–5) and underwent an average of 6 diagnostic tests (range, 1–12), including metabolic screening, repetitive imaging, single-gene testing and NGS (target or exome sequencing (ES)), before KUDP admission.

**Table 1:** Demographic data of the enrolled patients

	Number of patients (total n = 458)
Sex (male:female)	257:201
Mean age of symptom onset (years)	1.4 (range 0–41.9)
Mean age at first medical service (years)	1.9 (range, 0–57.9)
Mean age at KUDP admission (years)	6.2 (range, 0–58.7)
Number of diagnostic tests before the project (n, (%))	
< 5	99 (21.6%)
5–10	343 (74.9%)
> 10	16 (3.5%)
Time interval between symptom onset and the first medical evaluation (n, (%))	
< 1 month	264 (57.6%)
1–6 months	123 (26.9%)
7–12 months	26 (5.7%)
1–5 years	36 (7.9%)
> 5 years	9 (2.0%)
Time interval between the first medical evaluation and KUDP admission (n, (%))	
< 1 month	6 (1.3%)
1–6 months	53 (11.6%)
7–12 months	55 (12.0%)
1–5 years	213 (46.5%)
5–10 years	78 (17.0%)
> 10 years	53 (11.6%)

### Result of the KUDP

Enrolled patients underwent individualized tests by stage. The total numbers of performed tests were as follows: chromosomal microarray (CMA, 55 tests), single-gene testing (54 tests), target gene panel sequencing (57 tests), ES (413 tests), and nongenetic tests (4 tests). The results were provided to the referring clinician for clinical validation, and the final diagnosis was made based on the test results, the clinician’s opinion, and further validation if indicated. The overall final diagnostic rate was 52.8% (242 of 458 patients), and the yield was different for each group: 81.3% (26/32) for Group I, 53.3% (188/353) for Group II, and 38.4% (28/73) for Group III (Fig. 1). Detailed information on the diagnosed patients is presented in Additional file 1: Table 1. Among the patients who received a final diagnosis, 10 diagnoses were confirmed by CMA (10/242, 4.1%), 22 by a single gene test (22/242, 9.1%), 14 by a target gene panel (14/242, 5.8%), 193 by ES (193/242, 79.8%), and 3 by a nongenetic test (3/242, 1.2%). Simultaneous RNA sequencing was performed for 9 patients, and reanalysis of the ES data was performed for 48 patients, which resulted in an additional diagnosis for 7 patients (8/48, 16.7%). Final results were reported to the clinicians who initially referred the patients, clinical follow-up was conducted at regional hospitals and the center, and the availability of follow-up data varied. At the time of writing, 12 patients had started active treatment based on final diagnoses (nusinersen for 3 spinal muscular atrophy patients, a ketogenic diet for 2 GLUT1 deficiency syndrome cases, L-dopa for 2 dopa-responsive dystonia patients, acetylcholinesterase inhibitors for 2 congenital myasthenia cases, immunotherapy for 2 patients with myositis, and mexiletine therapy for a paramyotonia patient). Three families decided to withdraw life-sustaining treatment based on the diagnoses, and 3 patients ceased further genetic testing after the confirmation of nongenetic etiologies. All families received genetic counseling, and 16 families made plans for the next baby based on the counseling.

In 2020, the KUDP functional core laboratories which consisted of a laboratory for analyzing protein structure and function, two model organism screening centers (zebrafish and *Drosophila*), and preclinical science laboratories for metabolomics, stem cells, neuroscience and immunology was organized. All variants from ES data were reviewed based on previously reported findings, such as the function of genes, tissue expression, and previous experimental or clinical data. Variants in 40 genes were discussed at a monthly conference between the clinical expert consortium and functional core laboratories. Eighteen genes have been shared on the international matchmaking exchanger (MME) system to date, and functional studies have been completed or are in progress for 6 genes. Clinically, the KUDP has expanded to 2 independent programs, the child and adult KUDPs, since 2020. The aim of this separation was to focus on age-specific diseases and support underprivileged elderly individuals.

### Development of a web-based repository system

**Fig. 2:**
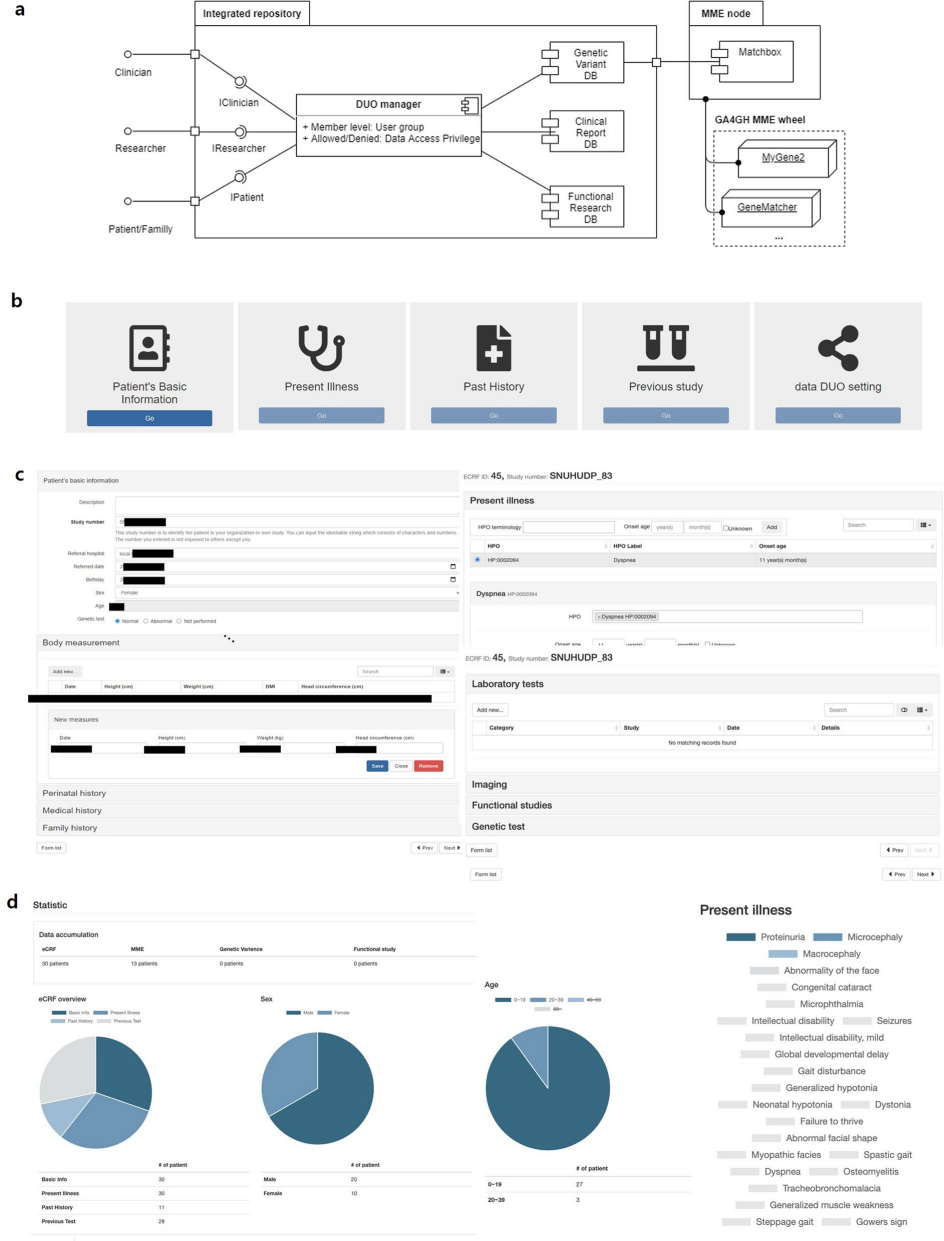
Overview of our system. **a** Each clinical and study dataset is stored in 3 databases (Clinical Report DB, Functional Research DB, and Genetic Variant DB). For data mediation and sharing, all stored data are controlled by data access privileges, which can be input by the data owner through the DUO manager. **b** The Clinical Report Database stores 4 types of clinical data, and the data can be shared and mediated by the DUO designated by the data owner. **c** The implemented interface of the Clinical Report database for data input. **d** Statistics website for our system showing the current data statistics for 30 patients according to each category (30 ‘basic patients’ information’, 30 ‘present illness’, 11 ‘past histories’ and 28 ‘previous tests’ records. The numbers in the figure originate from a trial version)

We are developing a web-based repository system to support cohort management and provide a data mediation function. Our system provides three databases for cohort management (Fig. 2a). The clinically screened data are stored and classified into 4 categories in the Clinical Report Database (Fig. 2b and c). The “basic patient information” category comprises general enrolled patient information, such as referring hospital, age, and sex. The significant symptoms that a patient is currently experiencing are included in the “present illness” category and defined as standardized terms with the Human Phenotype Ontology (HPO) project. Disease or genetic information that was previously provided from other hospitals and laboratories is stored in the past history and previous study categories.

The system also provides a mediation function based on a matchbox [12]. The matchbox is a standalone server to implement the MME protocol with standardized nomenclatures. We have applied this system and, through its use, implemented the connecting interface with two MME nodes (GeneMatcher and MyGene2) using their application programming interfaces (APIs) [13, 14]. The Data Usage Ontology (DUO) manager designates the access control privileges for the stored data as the standardized ontology structure [15]; through this, the data owner can manage and share their data for a designated period to designated users and groups only for a designated purpose. Figure 2d shows the statistics web page comprising the accumulated data in our system. This web page summarizes the registered data status according to a patient’s basic information. The introduced system is now in the final adjustment period. This system will be developed as a repository for rare disease data management in Korea.

## Illustrative cases

### Ending the diagnostic odyssey

A 21-year-old man was admitted to the KUDP for recurrent lymphedema, skeletal abnormalities, and dysmorphism. He was born at a gestational age of 35^+2^ weeks to nonconsanguineous healthy parents and admitted to the neonatal intensive care unit for fetal hydrops (diffuse subcutaneous edema, bilateral pleural effusion, and ascites). A pathological fracture was documented at the age of 2 months, and subsequent hearing loss, short stature, and macrocephaly arose. Clinicians suspected osteogenesis imperfecta type III and administered cyclic pamidronate before his teenage years. However, he did not have bluish sclerae or further fractures after discontinuing pamidronate. The patient also experienced recurrent pleural effusion, ascites, and bilateral hydrocele, requiring repeated aspiration since infancy. He presented short stature (162 cm at 17 years old, 3rd percentile), macrocephaly (55 cm, 97th percentile), and scoliosis. He had prognathism and bilateral dysplastic ears. A mild intellectual disability was confirmed. Multidisciplinary medical services, including several diagnostic tests and sustained supportive care, were provided, but he remained undiagnosed for 20 years until KUDP admission. Trio-ES demonstrated a heterozygous missense variant, c.2858G > A in *PIEZO1* (GRCh37, Chr16:88798876), the causative gene of the primary lymphatic malformation that was initially identified in 2015. We also identified a heterozygous small deletion (GRCh37, Chr16:88,782,477–88,876,207) by polymerase chain reaction (PCR) and Sanger sequencing [16].

### Nongenetic rare disease

The patient was a 6-year-old boy who presented with progressive muscle weakness. He started his diagnostic journey at 4 years old for a motor delay and elevated creatinine kinase levels (5245 IU/L, reference 20–270 IU/L). Subsequent genetic studies, a multiplex ligation-dependent probe amplification test and sequencing for *DMD*, a targeted gene panel for muscular dystrophy, and ES were conducted, but none of these tests resulted in a diagnosis. A muscle biopsy indicated many degenerating and regenerating fibers with endomysial fibrosis, consistent with muscular dystrophy, but the immunohistochemical study showed normal dystrophin expression. After KUDP admission, the expert consortium reviewed the patient’s entire medical history and noticed rapid deterioration and weakness in the lower extremities within a year, followed by progression to the upper extremities after influenza infection. The patient became dependent on a wheelchair and could not elevate his arms within 2 years of symptom recognition. We suspected a rare form of inflammatory myositis and checked for multiple myositis-specific autoantibodies. As a result, he was diagnosed with anti-signal recognition particle antibody-related inflammatory myositis. He was treated with corticosteroids, immunoglobulin and rituximab after diagnosis and showed improvement in muscle power.

### Identification of a case of dual diagnosis

The third patient was 35 months old when he was admitted to the KUDP. He was born at a gestational age of 40 weeks following an uneventful pregnancy. He had multiple café-au-lait spots that spread over time. His developmental milestones were slightly delayed, and he showed tiptoeing followed by left ankle contracture since the age of 30 months. He visited the tertiary center and underwent magnetic resonance imaging (MRI) of the brain and target gene panel sequencing. The brain MRI indicated high signal intensity lesions on the bilateral basal ganglia. The ophthalmological examination demonstrated bilateral Lisch nodules. A pathogenic variant, c.344C > T, in *NF1* was identified by a target gene panel. The patient was diagnosed with neurofibromatosis type 1 (NF1) with atypical NF1 stigmata on brain imaging, and rehabilitation was recommended for abnormal walking. His parents voluntarily visited the KUDP center for further evaluation due to his sustained gait abnormality. The patient showed increased muscle tone and definite upper motor neuron signs in the lower legs, and a review of the MRI suggested a metabolic disorder such as Leigh disease instead of NF1 stigmata, although NF1 was also consistent with the phenotype. We enrolled the patient and performed additional mitochondrial genome sequencing, which revealed the homoplasmy variant, 3697G > A in MT-ND, known as the causative variant for Leigh syndrome [17].

### Emergent process for an early medical decision

A 2-month-old girl who showed intractable seizures, hyperreflexia, and hypertonia since her first day of life was referred to the KUDP. She was administered multiple antiepileptic drugs and muscle relaxants, including continuous intravenous midazolam, and was fully dependent on a mechanical ventilator. Serial brain MRI revealed rapidly progressing brain atrophy. The expert consortium classified her as an emergent case requiring rapid diagnosis and clinical decision-making. Rapid ES was conducted on the family, and compound heterozygous variants, c.1276C > T (GRCh37, Chr7:2580977) and c.1313_1314delAG (GRCh37, Chr7:2580938), from *BRAT1* were identified within 2 weeks. The patient was confirmed to have rigidity and multifocal seizure syndrome (MIM#614498). The parents withdrew life-sustaining treatment after the diagnosis and comprehensive genetic counseling. Two years later, the couple had a healthy baby after undergoing prenatal genetic testing.

## Discussion

Phase I of the KUDP aimed to solve the unmet needs of PLWRDs and establish infrastructures for long-term research on RDs in Korea. In this study, we identified the remaining unmet needs of rare disease patients. Despite easy access to tertiary hospitals, enrolled patients remained undiagnosed for approximately 5 years. The total diagnostic yield was quite high (52.8%), but the yield varied between groups. Over 80% of Group I patients received a final diagnosis, whereas only one-third of patients from Group III received genetic confirmation. As the number of patients classified as Group I decreased over the years and that classified as Group III increased, the annual diagnostic yield slightly decreased each year (57.7% in 2018, 46.3% in 2019, and 52.0% in 2020). We expect a rise in the number of patients classified as Group III, a more appropriate group for the purposes of the KUDP, and that these patients will be the major population of the KUDP. There are many different national programs for RDs, which have various inclusion criteria and protocols. Their diagnostic yields vary from 28% in the UDN to 67% in SpainUDP [7, 8, 18, 19]. Unlike other national programs, the KUDP and UDN applied single-gene or nongenetic tests. Approximately 15% of diagnosed patients from both programs were confirmed by direct clinical testing, such as CMA, or clinical rounds. This finding suggests that comprehensive phenotyping by experts should be performed before up-to-date tests to guide best practices. We also expect new gene discoveries with the collaboration of functional core laboratories launched in 2020, similar to that achieved in the UDN, FORGE and IRUD [19–21].

We discussed the enrollment criteria annually, and the “appropriate test” standard changed as the government insurance policy changed permitted diagnostic tests. CMA and target gene panel sequencing were approved in the second half of 2019. Some single-gene tests became available at official laboratories over time. These tests were frequently conducted for enrolled patients before early 2019 but were rarely performed as they became “appropriate tests” after 2019. The standard will change if ES or other tests officially become available at the clinic. Undiagnosed patients who already underwent ES represent RD patients with a major unmet need. We planned regular clinical follow-ups for the patients and reanalyzed ES data every 6 months to 1 year. We screened and selected patients with the following criteria: (1) patients highly suspected to have a certain genetic disease with characteristic features; (2) patients with a pathogenic or likely pathogenic variant of one allele in the autosomal recessive pattern gene and the matched phenotypes; and (3) patients with a rapidly deteriorating disease course without known causes. Among the 48 reanalyzed cases, 8 patients were definitively diagnosed, and functional validation for one candidate gene is ongoing.

**Fig. 3:**
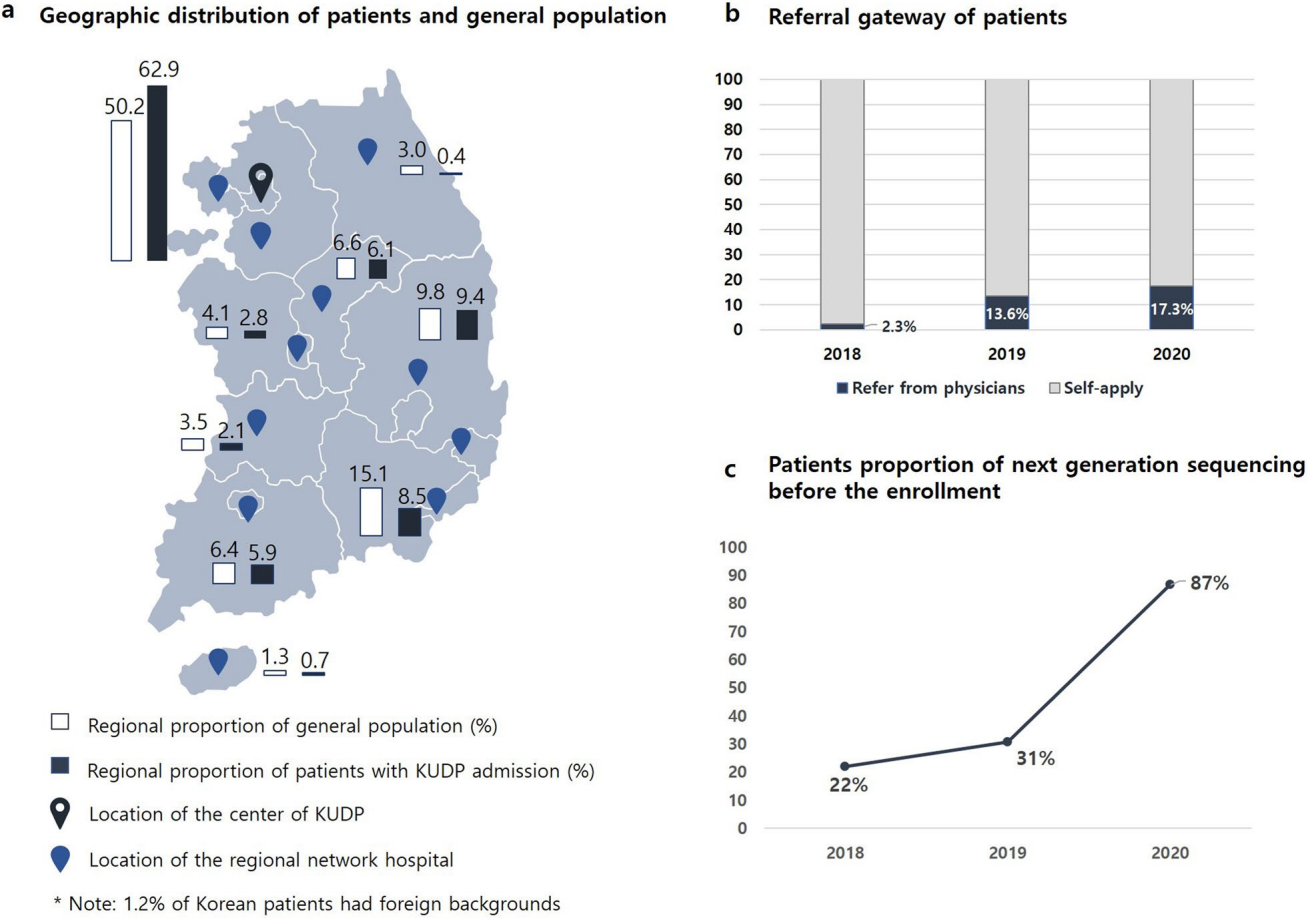
Summary of the social data. **a** Patients nationwide were enrolled in the Korean Undiagnosed Diseases Program (KUDP) and showed a similar regional proportion compared with the general population. **b** The number of referrals from physicians increased every year. **c** The number of patients who underwent next-generation sequencing before enrollment increased every year

The basic information of the patients revealed weaknesses in the Korean medical system. The Korean government provides universal medical insurance that prioritizes easy accessibility to above-average medical services and determines the affordable cost and indication of all medical practices. Patients can choose tertiary hospitals for their first medical service and visit several hospitals at the same time, regardless of the stepwise medical delivery system. Many patients had same tests at different hospitals and waste resources. The current medical system assures patient rights and a certain level of medical services among the general population, but it also has serious problems in the context of PLWRD. For this reason, applications for the KUDP were conducted in two ways: referral from a clinician or a voluntary visit to the coordinating central hospital. The latter accounted for the majority of the total enrollment since the KUDP pilot project [11]. The KUDP tried to establish a nationwide network and improve the medical delivery system. We introduced the KUDP in various academic societies and regional rare disease network centers nationwide, requesting early referrals followed by referrals back with exact diagnoses and therapeutic guides within the medical network, preventing patients from having to go from hospital to hospital. The Korea Disease Control and Prevention Agency designated regional rare disease centers in each province and established a nationwide rare disease network in 2019. We also provided various channels for patient referrals, such as by phone, electronic mail, and the internet. As a result, the program recruited patients throughout the country (Fig. 3a), and the proportion of referrals from regional network hospitals dramatically increased over the 3 years (Fig. 3b) [22].

As only target gene panels consisting of a limited number of genes are permitted in the Korean insurance system to date, further approaches, such as ES, are only performed by research platforms. Such data, however, seemed hard to qualify and manage consistently. The entire proportion of patients who underwent NGS before admission was 38.6% (177/458), and this proportion dramatically increased in 2020 (Fig. 3c); their diagnostic yield was 48.0% (85/177), similar to the overall yield. Among them, 2 had typical clinical features and were diagnosed by a single gene test (*DYT1* and *SMN1*). ES was repeated for 150 patients, and 49 patients received an additional diagnosis. Some identified genes were recently documented as causes of Mendelian disorders; however, others were not. Although we could not compare the exact entire dataset because of data unavailability, we suggest that appropriate clinical assessments might allow further diagnoses from the same data. We noted a male patient with severe developmental delay, increased T3 levels, and family history evident of X-linked disorder. He remained undiagnosed after ES but was confirmed to have a pathogenic *SLC16A2* variant with low sequence depth. We noted a somatic variant in *PIK3CA* based on clinical suspicion in an undiagnosed patient with hemihypertrophy, macrocephaly, and intellectual disability. In addition, 2 patients had nongenetic diseases (juvenile dermatomyositis and anti-signal recognition particle antibody myositis) after a year of diagnostic journey. Detailed phenotyping and clinical experience in ultrarare diseases are the first steps for evaluating PLWRD, indicating the importance of RD experts and such programs. Our findings also highlighted the necessity of qualified data management and planned regular reanalysis in the long term.

The development of web-based systems was another goal of the KUDP. The system included electronic cohort management based on the HPO and searching or selective data sharing functionalities for open electronic case reports, MME, and functional research. This aspect of the project is in the final adjustment period, and we expect this system to facilitate clinical data sharing among clinicians, avoid unnecessary data generation, and promote international collaboration. We also developed functional core laboratories for validating candidate genes or variants originating from the KUDP, which are expected to facilitate independent work. The data management system and functional core laboratories will be essential infrastructure for sustainable research on rare diseases in Korea. The program will clinically focus on Group III patients and lead to functional research followed by new gene/disease discovery to determine disease pathomechanisms and treatment targets.

## Conclusions

We have summarized the results of Phase I of the KUDP. We successfully conducted Phase I of the KUDP for 3 years with favorable clinical outcomes and affirmed the unmet needs of RD patients, such as issues arising from a prolonged diagnostic journey, unavailable or limited tests, and the absence of standard strategies for preexisting NGS data. Infrastructure for sustainable program, a web-based integrated system and functional core laboratories, was established and have started to progress. They will play essential roles in the future of the KUDP.

## Methods

### Project design and study approval

The KUDP project was initiated and supported by the Korea Disease Control and Prevention Agency. Seoul National University Children’s Hospital functioned as the main center and supervised the entire clinical process. KUDP applications were made by direct referral or referral letters from regional network hospitals and through primary screening of self-visiting patients at the central hospital. The study protocol, including the biorepositories, diagnostic procedures, clinical data collection, selective functional experiments and web-based data sharing, was approved by the Institutional Review Board (IRB) of Seoul National University Hospital (IRB No. 1904-054-102).

### Enrollment criteria and classification

Patient screening and enrollment were performed by the KUDP expert consortium, comprising clinical experts from various clinical departments, including neurology, immunology, nephrology, cardiology, orthopedics, ophthalmology and laboratory medicine. Enrolled patients were required to meet one of the following criteria: (1) undiagnosed after appropriate tests conducted by experts; (2) suspected to have a medically actionable disease that presented rapid deterioration and an irreversible clinical course; or (3) had a diagnostic journey of more than 5 years despite regular check-ups at second or tertiary centers. Enrolled patients were classified into 3 groups. Group I was defined as patients who suspected to have a specific diagnosis which can be documented by direct testing as a result of clinical perception and basic medical information. Patients diagnosed with certain disease categories, usually with genetic or phenotypic heterogeneity, were defined as Group II (e.g., epileptic encephalopathy, common variable immunodeficiency). Group III was defined as patients with an uncategorized or atypical disease. Most patients requiring diagnostic tests covered by insurance systems at the time of screening were excluded and recommended to undergo routine diagnostic processes at their referring hospital. The consortium discussed each case, decided which diagnostic tests should be performed, and reviewed the test results.

### Diagnostic workflow and follow-up process

**Fig. 4:**
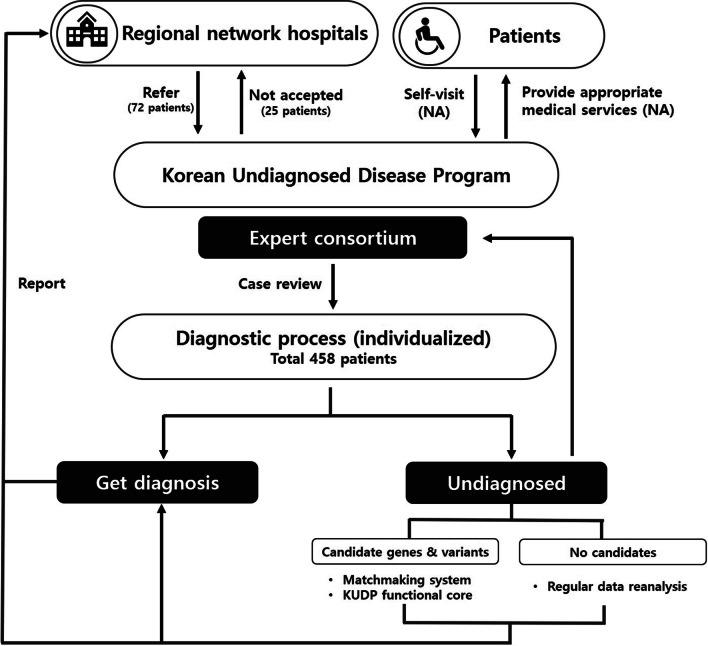
Schematic workflow of the Korean Undiagnosed Diseases Program. Clinicians or patients could apply for the program. The expert consortium decided on the entire diagnostic process for each enrolled patient and communicated closely with the functional core laboratories for variant validation and matchmaking

Process decisions and operations were made by the KUDP expert consortium (Fig. 4). All data generation and analyses were centralized at the coordinating center, and the results were shared with the regional network hospitals. All patients were discussed at an expert consortium and had sequential diagnostic tests, from nongenetic testing to NGS or RNA sequencing. Data were reviewed, and the expert consortium made a final diagnosis based on the test results. If a patient did not obtain a confirmative diagnosis after ES, the consortium discussed the validity of further evaluation, including RNA sequencing, whole-genome sequencing and other biochemical testing. The patients had regular check-ups at center or network hospitals, and the reanalysis of ES data was conducted for some patients based on revised phenotypes and updated analytic pipelines. Possible candidate genes or variants were discussed with the KUDP functional core laboratory, followed by independent validation or international sharing by a matchmaking system.

## Supplementary Information

**Additional file 1.** Detailed information of genetically confirmed patients.

## Data Availability

All data generated or analyzed for the study are available from the corresponding author upon reasonable request.

## Abbreviations

RD: Rare disease
PLWRD: People living with rare diseases
NGS: Next-generation sequencing
KUDP: Korean undiagnosed diseases program
CMA: Chromosomal microarray
ES: Exome sequencing
MME: Matchmaking exchanger
HPO: Human phenotype ontology
DUO: Data usage ontology
MRI: Magnetic resonance imaging
NF1: Neurofibromatosis type 1

## Declarations

### Prior presentation

A non-peer-reviewed version of this article has been posted on a pre-print server since 24 Feb 2022 at ResearchSquare. (https://doi.org/10.21203/rs.3.rs-1371884/v1. Licensed under a CC BY 4.0 License).
